# Supplementary material for: Vessel noise levels drive behavioural responses of humpback whales with implications for whale-watching
Source: eLife. 2020 Jun 16;9:e56760. doi: 10.7554/eLife.56760 (PMC7324156; doi:10.7554/eLife.56760)
Supplement: Supplementary file 1. — Ethogram for surface behavioural events and conspicuous underwater events, for both instantaneous and continuous events, on a resting ground. [file elife-56760-supp1.docx]

**Supplementary 1.** Humpback whale behavioural ethogram. Ethogram for surface behavioural events and conspicuous underwater events, for both instantaneous and continuous events, on a resting ground.

| **Behavioural event** | **Definition** | **Event** |
| --- | --- | --- |
| Breach | Whale leaps head first mostly clear of the water. An umbrella term for a true breach and half breach (aka ‘belly flop’). Generally, the whale takes a breath during a breach, so a respiration was also recorded during each breach. | Instantaneous |
| Spy hop | Whale raises its head vertically out of the water. | Instantaneous |
|  |  |  |
| Peduncle dive | Whale submerges exposing the peduncle but not the fluke (aka ‘peduncle arch dive’, ‘round out’). | Instantaneous |
| Fluke dive | Whale performs a fluke-up or fluke-down dive. | Instantaneous |
| Fluke thrash | Whale moves fluke rapidly through the water in a sideways movement, which is commonly an underwater movement (aka ‘fluke swish’, ‘fluke cock’, ‘fluke slash’). | Instantaneous |
| Fluke slap | Whale slaps the surface of the water forcefully with the ventral or dorsal side of its fluke/tail (aka ‘lobtail’, ‘tail slap’). | Instantaneous |
| Peduncle throw | Whale strikes the surface of the water with the lateral side of the peduncle and fluke (aka ‘peduncle slap’, ‘tail breach’). | Instantaneous |
|  |  |  |
| Bubble stream | Underwater stream of bubbles from the blow hole. | Instantaneous |
|  |  |  |
| Head slap | Whale strikes the surface of the water with its head, in any direction (aka ‘chin slap’). | Instantaneous |
| Head lunge | Fast upward thrusting of head forward out of the water at an angle <40**°**. | Instantaneous |
| Roll | Whale rolls on its back or side, with pectoral fins in the air. | Instantaneous |
| Pectoral slap | Whale slaps the surface of the water with its pectoral fin (aka ‘flippering’, ‘flipper slap’). | Instantaneous |
|  |  |  |
| Surface travel | Whale swims with part of the body breaking the water surface for longer periods than a typical surfacing. | Continuous |
| Logging | Whale remains on the surface with no other behavioural event visible for more than 15 sec. Logging behaviour classified as a resting state. | Continuous |
