# Supplementary material for: Vessel noise levels drive behavioural responses of humpback whales with implications for whale-watching
Source: eLife. 2020 Jun 16;9:e56760. doi: 10.7554/eLife.56760 (PMC7324156; doi:10.7554/eLife.56760)
Supplement: Supplementary file 2. — Models were linear mixed effects models (LMM) and penalized quasi-likelihood generalised liner mixed models (GLMM-PQL). Interaction of fixed effects = treatment*phase. Random effect = (1|Individual). Weights = the duration of time for each phase e.g. more weight will be given to longer phases. Corr = to account for temporal auto-correlation within follows, the model was used with an auto-regressive structure with lag one. REML = restricted maximum likelihood estimation. † Fitted for both mother and calf. [file elife-56760-supp2.docx]

**Supplementary 2.** Mixed models used in analyses to test for behavioural effects of underwater vessel noise on humpback whales. Models were linear mixed effects models (LMM) and penalized quasi-likelihood generalised liner mixed models (GLMM-PQL). Interaction of fixed effects= treatment*phase. Random effect= (1|Individual). Weights = the duration of time for each phase e.g. more weight will be given to longer phases. Corr = to account for temporal auto-correlation within follows, the model was used with an auto-regressive structure with lag one. REML= restricted maximum likelihood estimation. † Fitted for both mother and calf.

| **Model** | **Model details** | **Model** | **Error distribution** | **Link function** |
| --- | --- | --- | --- | --- |
| 1 | Proportion.time.logging ~ treatment*phase + (1\|Individual), weights† | GLMM-PQL | Binomial | Logit |
| 2 | Presence.inst.beh.events (1/0) ~ treatment*phase + (1\|Individual), weights† | GLMM-PQL | Binomial | Logit |
| 3 | Respiration.rate (breaths min^-1^) ~ treatment*phase + (1\|Individual), method="REML"† | LMM | NA | NA |
| 4 | Mean.heading.change (degrees) ~ treatment*phase + (1\|Individual), method="REML"† | LMM | NA | NA |
| 5 | Mean.swim.speed (m s^-1^) ~ treatment*phase + (1\|Individual), corr, method="REML"† | LMM | NA | NA |
