# Supplementary material for: Vessel noise levels drive behavioural responses of humpback whales with implications for whale-watching
Source: eLife. 2020 Jun 16;9:e56760. doi: 10.7554/eLife.56760 (PMC7324156; doi:10.7554/eLife.56760)
Supplement: Supplementary file 3. — Broadband source levels (SL) of vessel noise treatments produced during controlled exposure experiments, and the perceived received levels (RL, mean ± SD) during calibrations in 14 m water depth. The SL of the research vessel was 140 ± 2 dB re 1μPa @1 m. Broadband ambient noise was 103 dB re 1 μPa (18 August 2018). [file elife-56760-supp3.docx]

**Supplementary 3.** Perceived received levels of vessel noise by focal whales. Broadband source levels (SL) of vessel noise treatments produced during controlled exposure experiments, and the perceived received levels (RL, mean ± SD) during calibrations in 14 m water depth. The SL of the research vessel was 140±2 dB re 1μPa @1m. Broadband ambient noise was 103 dB re 1 μPa (18 August 2018).

| **Treatment** | **Palyback SL dB re 1 μPa @ 1 m** | **Vessel status** | **RL dB re 1μPa** |
| --- | --- | --- | --- |
| Control | 124 | Transiting 800 rpm @ 22m | 109 |
| Low | 148 | Stationary @ 88m | 111-112 (111 ± 0) |
| Medium | 160 | Stationary @ 88m | 121-123 (122 ± 1) |
| High | 172 | Stationary @ 88m | 132-134 (133 ± 1) |
