# Supplementary material for: Vessel noise levels drive behavioural responses of humpback whales with implications for whale-watching
Source: eLife. 2020 Jun 16;9:e56760. doi: 10.7554/eLife.56760 (PMC7324156; doi:10.7554/eLife.56760)
Supplement: Transparent reporting form [file elife-56760-transrepform.pdf]

eLIFE
1st Floor
24 Hills Road
Cambridge CB2 1JP, UK
P 01223 855340
W elifesciences.org
T @elife

eLife's transparent reporting form

We encourage authors to provide detailed information *within their submission* to facilitate the interpretation and replication of experiments. Authors can upload supporting documentation to indicate the use of appropriate reporting guidelines for health-related research (see EQUATOR Network), life science research (see the BioSharing Information Resource), or the ARRIVE guidelines for reporting work involving animal research. Where applicable, authors should refer to any relevant reporting standards documents in this form.

If you have any questions, please consult our Journal Policies and/or contact us: editorial@elifesciences.org.

Sample-size estimation

- You should state whether an appropriate sample size was computed when the study was being designed
- You should state the statistical method of sample size computation and any required assumptions
- If no explicit power analysis was used, you should describe how you decided what sample (replicate) size (number) to use

Please outline where this information can be found within the submission (e.g., sections or figure legends), or explain why this information doesn't apply to your submission:

Experiments: Controlled exposure experiments were conducted on resting humpback whale mother-calf pairs that were logging at the surface, on a breeding ground. The breeding ground, Exmouth Gulf, is a remote region dominated by biological sounds with minimal noise from anthropogenic activities, such as vessels, thus allowing for an ideal study area for noise playback experiments. Controlled exposure experiments consisted of a *before, during* and *after* experimental design, where the behaviour of the whales was recorded through focal follows. Mother-calf pairs were used as they offer a standardised behaviour that facilitates detection of noise induced disturbance.

Sample size (n) to test the hypothesis: Field experiments are limited by time, money, or logistics, thus pilot studies for a power analyses are generally not feasible. However, our study has resulted in the largest sample size, to our knowledge, for controlled exposure experiments (aka. Behavioural response studies) examining vessel noise (n = 42). For each treatment (control/low, medium and high noise), over 10 replicates were conducted to ensure sufficient power for analyses (Dunlop et al. 2012). Dunlop et al. (2012) stated for behavioural response studies with noise playbacks that "The power-analysis graph (Fig. 1) generated for the response to tones indicates that a sample size of 10 per treatment (that is 10 controls and 10 exposures) was needed to detect a significant change in course traveled [of whales]."

Dunlop RA, Noad MJ, Cato DH (2012) Behavioral-response studies: problems with statistical power. The effects of noise on aquatic life. Springer. Pages 293-297.

Stated in the manuscript: "For CEEs, ten replicates of each treatment were aimed to be conducted to ensure sufficient power for analyses."

Replicates

- You should report how often each experiment was performed
- You should include a definition of biological versus technical replication

eLife Sciences Publications, Ltd is a limited liability non-profit non-stock corporation incorporated in the State of Delaware, USA, with company number 5030732, and is registered in the UK with company number FC030576 and branch number BR015634 at the address 1st Floor, 24 Hills Road, Cambridge CB2 1JP | August 2014

- The data obtained should be provided and sufficient information should be provided to indicate the number of independent biological and/or technical replicates
- If you encountered any outliers, you should describe how these were handled
- Criteria for exclusion/inclusion of data should be clearly stated
- High-throughput sequence data should be uploaded before submission, with a private link for reviewers provided (these are available from both GEO and ArrayExpress)

Please outline where this information can be found within the submission (e.g., sections or figure legends), or explain why this information doesn't apply to your submission:

Replicates: After data filtering, focal follows that were used in analyses included 13 control (including 4 low), 14 medium and 15 high noise replicates. Control and low noise treatments were pooled as the received levels of vessel noise were around ambient noise.

Whale ID: To ensure samples were independent, the same mother-calf pair was never sampled twice. To identify individuals, photo-identification of the dorsal fin shape using a DSLR (Canon 50D 400 mm lens) and aerial photographs of the dorsal side using an unmanned aerial vehicle (UAV) were taken to create a photo-identification catalogue. The catalogue was taken on the boat to double-check individuals for the shape of their dorsal fin and patterns/markings prior to CEEs.

Playback noise: from the 60 min modified vessel noise recordings, we extracted 15 different 15 min sound files at random times in the recording, for each low, medium and high noise files (i.e. 45 noise files, and a control noise). To avoid pseudoreplication, each recording for control, low, medium and high were selected at random for each consecutive CEE using the function *randperm* in MATLAB (The MathWorks, Inc., Natick, MA, USA).

Replicates included: Replicates of CEEs were conducted >3 km from any previous exposure experiment the same day. Focal follows were only conducted during good weather conditions; <15 knot winds, Beaufort sea state <3 and no precipitation. Replicates were included in analyses if an experiment was composed of three scenarios (*before, during, after*) on the same focal whale (i.e. repeated measures). For these filtered CEEs, there was 29.4 hours of focal follow data which were conducted in daylight hours between 7:20 to 18:20. The mean duration for *before* flights was 13:09 mins (0.003 SD), *during* was 15:04 (0.002 SD) mins and *after* was 14:48 (0.004 SD) mins. The closest point of approach to the focal pair was measured on average at 135.3 m (56.0 SD). Filtered CEE included 42 mother-calf pairs, and were conducted in water temperatures between 19-24 ºC, in water depths between 13-21 m (mean = 17 m).
Replicates not included: Data were filtered, and CEEs that ceased early and/or were not used in analyses were due to i) identifying repeat whales (*n*= 2), ii) if a boat approached close by (*n*= 5), iii) if the sun was setting and did not allow sufficient time to complete the CEE (*n*= 1), iv) if conspecifics arrived <100 m to the focal pair (*n*= 12), v) if the vessel ended up approaching the whale too close (e.g. 70 m, *n*= 1), vi) if the whales possibly reacted to a loud gear-shift in the *during* phase (*n*= 2), vii) if the whales were predominantly slow travelling in the *before* phase (*n*= 15), viii) due to technical issues (*n*= 4), ix) if the weather deteriorated (*n*= 1), and x) if other species interacted with the whales appearing to cause behavioural changes (e.g. a school of fish, *n*= 1; silver gulls pecking the skin of the whale, *n*= 6). The remaining CEEs that had *before*, *during* and *after* data on the same mother-calf pair were used in analyses.

Statistical reporting

- Statistical analysis methods should be described and justified
- Raw data should be presented in figures whenever informative to do so (typically when N per group is less than 10)
- For each experiment, you should identify the statistical tests used, exact values of N, definitions of center, methods of multiple test correction, and dispersion and precision measures (e.g., mean, median, SD, SEM, confidence intervals; and, for the major substantive results, a measure of effect size (e.g., Pearson's r, Cohen's d)
- Report exact p-values wherever possible alongside the summary statistics and 95% confidence intervals. These should be reported for all key questions and not only when the p-value is less than 0.05.

Please outline where this information can be found within the submission (e.g., sections or figure legends), or explain why this information doesn't apply to your submission:

See methods section, "Data analyses for the behavioural effects on whales", and supplementary table 2. Mixed models used in analyses to test for effects of underwater vessel noise on humpback whales.

(For large datasets, or papers with a very large number of statistical tests, you may upload a single table file with tests, Ns, etc., with reference to sections in the manuscript.)

Group allocation

- Indicate how samples were allocated into experimental groups (in the case of clinical studies, please specify allocation to treatment method); if randomization was used, please also state if restricted randomization was applied
- Indicate if masking was used during group allocation, data collection and/or data analysis

Please outline where this information can be found within the submission (e.g., sections or figure legends), or explain why this information doesn't apply to your submission:

Methods section: "From the 60 min modified vessel noise recordings, we extracted 15 different 15 min sound files at random times in the recording, for each low, medium and high noise files (i.e. 45 noise files, and a control noise). To avoid pseudoreplication, each recording for control, low, medium and high were selected at random for each consecutive CEE using the function *randperm* in MATLAB (The MathWorks, Inc., Natick, MA, USA)."

Additional data files ("source data")

- We encourage you to upload relevant additional data files, such as numerical data that are represented as a graph in a figure, or as a summary table
- Where provided, these should be in the most useful format, and they can be uploaded as "Source data" files linked to a main figure or table
- Include model definition files including the full list of parameters used
- Include code used for data analysis (e.g., R, MatLab)
- Avoid stating that data files are "available upon request"

Please indicate the figures or tables for which source data files have been provided:

playback vessel noise of the research vessel and how it relates to whale-watch vessels. Thus, enabling the manuscript to provide recommendations of relevance to whale-watching guidelines.

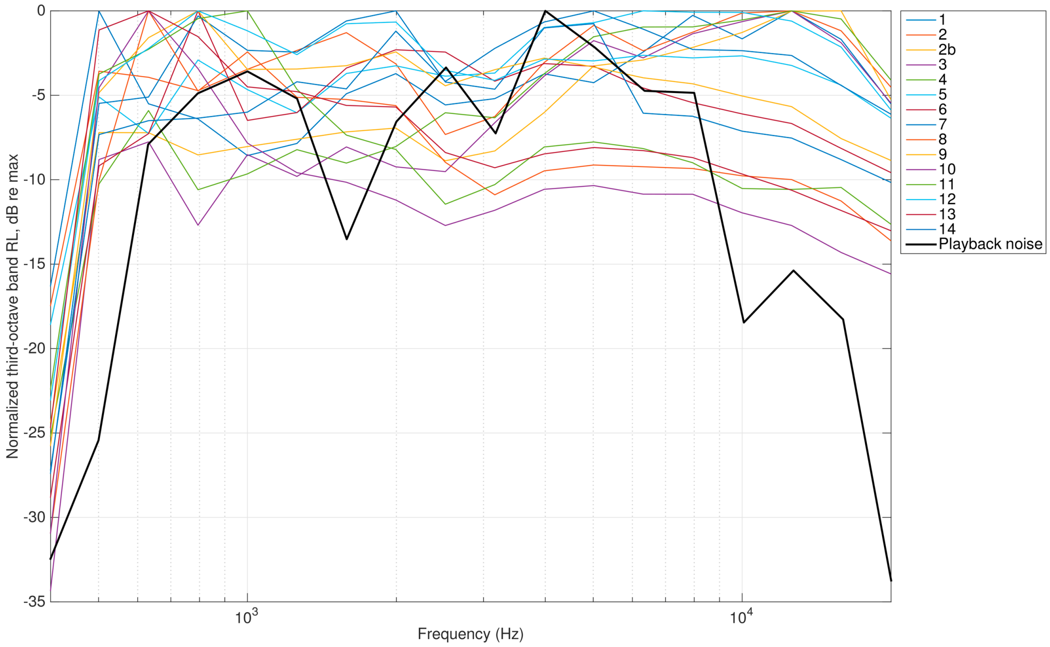

Figure 1- figure supplement 2. Spectral signatures of whale-watch vessel noise (*n*= 14) and research vessel playback noise (bold) in third octave-bands from 400 Hz to 20 kHz, normalised on a dB scale. Whale-watch vessels (10-22 m in length) recorded at slow speed (≤8 kn) in Exmouth Gulf, Australia, and Tenerife, Canary Islands, representing sailing motor (*n*= 4), catamaran (*n*= 5) and motor vessels (*n*= 5) as a part of a separate project (Arranz, P. et al. unpublished data). All third octave-bands have been subjected to a steep 400 Hz high pass filter to mimic the Lloyd mirror effect on a resting whale at the surface.
